# Distinct Regulatory Effects of Myeloid Cell and Endothelial Cell NAPDH Oxidase 2 on Blood Pressure

**DOI:** 10.1161/CIRCULATIONAHA.116.023877

**Published:** 2017-05-30

**Authors:** Can Martin Sag, Moritz Schnelle, Juqian Zhang, Colin E. Murdoch, Sabine Kossmann, Andrea Protti, Celio X.C. Santos, Greta Sawyer, Xiaohong Zhang, Heloise Mongue-Din, Daniel A. Richards, Alison C. Brewer, Oleksandra Prysyazhna, Lars S. Maier, Philip Wenzel, Philip J. Eaton, Ajay M. Shah

**Affiliations:** From King’s College London British Heart Foundation Centre of Excellence, Cardiovascular Division, United Kingdom (C.M.S., M.S., J.Z., C.E.M., A.P., C.X.C.S., G.S., X.Z., H.M.-D., D.A.R., A.C.B., A.P., P.J.E., A.M.S.); Klinik und Poliklinik für Innere Medizin II, Universitätsklinikum Regensburg, Regensburg, Germany (C.M.S., L.S.M.); Department of Cardiology and Pneumology, Medical Center Goettingen, Germany (M.S.); and Center for Cardiology and Center for Thrombosis and Hemostasis, University Medical Center Mainz, Germany (S.K., P.W.).

**Keywords:** angiotensin II, blood pressure, mice, NADPH oxidase

## Abstract

**Background:**

Hypertension caused by increased renin-angiotensin system activation is associated with elevated reactive oxygen species production. Previous studies implicate NADPH oxidase (Nox) proteins as important reactive oxygen species sources during renin-angiotensin system activation, with different Nox isoforms being potentially involved. Among these, Nox2 is expressed in multiple cell types, including endothelial cells, fibroblasts, immune cells, and microglia. Blood pressure (BP) is regulated at the central nervous system, renal, and vascular levels, but the cell-specific role of Nox2 in BP regulation is unknown.

**Methods:**

We generated a novel mouse model with a floxed Nox2 gene and used Tie2-Cre, LysM Cre, or Cdh5-CreERT2 driver lines to develop cell-specific models of Nox2 perturbation to investigate its role in BP regulation.

**Results:**

Unexpectedly, Nox2 deletion in myeloid but not endothelial cells resulted in a significant reduction in basal BP. Both Tie2-CreNox2 knockout (KO) mice (in which Nox2 was deficient in both endothelial cells and myeloid cells) and LysM CreNox2KO mice (in which Nox2 was deficient in myeloid cells) had significantly lower BP than littermate controls, whereas basal BP was unaltered in Cdh5-CreERT2 Nox2KO mice (in which Nox2 is deficient only in endothelial cells). The lower BP was attributable to an increased NO bioavailability that dynamically dilated resistance vessels in vivo under basal conditions without a change in renal function. Myeloid-specific Nox2 deletion had no effect on angiotensin II–induced hypertension, which, however, was blunted in Tie2-CreNox2KO mice, along with preservation of endothelium-dependent relaxation during angiotensin II stimulation.

**Conclusions:**

We identify a hitherto unrecognized modulation of basal BP by myeloid cell Nox2, whereas endothelial cell Nox2 regulates angiotensin II–induced hypertension. These results identify distinct cell-specific roles for Nox2 in BP regulation.

## Abstract 2

Supplemental Digital Content is available in the text.

The renin-angiotensin system plays a central role in blood pressure (BP) regulation, and its long-term activation contributes to hypertension. Both animal and human studies have implicated increased reactive oxygen species (ROS) production in the pathophysiology of angiotensin II (AngII)–dependent hypertension.^1,2^ ROS have complex cell-, source-, and context-specific roles, ranging from physiopathological redox signaling to inactivation of nitric oxide (NO) and detrimental oxidation of cellular biomolecules.^3,4^ In experimental models, ROS scavengers can attenuate the hypertensive response to AngII,^1^ but randomized clinical trials of general antioxidant approaches have failed to demonstrate a reduction in cardiovascular morbidity and mortality.^5^ Such global inhibition approaches may affect beneficial redox signaling as well as detrimental oxidative stress. A better understanding of the roles of ROS in BP regulation and hypertension is therefore necessary to develop novel and more refined therapeutic approaches.

NADPH oxidase (Nox) family proteins are major sources of ROS in the cardiovascular system and are important in redox signaling.^6,7^ They contain a Nox catalytic subunit that mediates ROS generation through electron transfer from NADPH to molecular oxygen. Five different oxidases, each based on a distinct Nox catalytic subunit (Nox1–5), have been identified. They have tissue-specific distribution and differing modes of activation based on their varying requirements for accessory subunits. Different Nox isoforms may have distinct roles even in the same cell type, thought to be related to their coupling to different intracellular signaling pathways or the production of different ROS (superoxide versus hydrogen peroxide).^6^ This is relevant from a therapeutic perspective because isoform-selective Nox inhibitors are currently being developed.^8^ Previous work suggested an involvement of Nox1 in the genesis of AngII-dependent hypertension. Mice globally deficient in p47^phox^, a subunit required for both Nox1 and Nox2 function, display a reduced hypertensive response to AngII,^9^ as do global Nox1 knockout (KO) mice,^10^ whereas vascular smooth muscle–targeted Nox1 transgenic mice develop exaggerated AngII-induced hypertension.^11^ The role of Nox2 is less clear. This isoform is expressed in endothelial cells, fibroblasts, cardiomyocytes, inflammatory cells, and microglia,^6^ sites that are of interest given that BP is regulated at the central nervous system, renal, vascular, and cardiac levels. Nox2 is involved in the genesis of endothelial dysfunction in diverse models.^6,7^ It is interesting to note that ROS production in the subfornical organ in the brain is implicated in the vasopressor effects of AngII,^12^ and the knockout of p22^phox^, a subunit required for Nox1, Nox2, and Nox4 function, at this site blunted AngII-induced hypertension.^13^ p47^phox^ KO mice have reduced renal afferent arteriolar constrictor responses to AngII,^14^ and the hypertensive effect of AngII has been shown to involve infiltration of the vasculature by p47^phox^-containing T lymphocytes^15^ and Nox2-competent monocytic cells.^16^ Although these studies suggest an involvement of Nox proteins in AngII-dependent hypertension, they do not directly establish the role of Nox2, in particular its potential cell-specific role. In this study, we have used a novel mouse model with a floxed Nox2 gene to study the role of endothelial and myeloid cell Nox2 in BP regulation. Unexpectedly, we found that myeloid cell Nox2 has an essential role in the basal regulation of BP, whereas activation of endothelial Nox2 contributes to AngII-dependent hypertension.

## Methods

### Generation of Mice With a Floxed Nox2 Allele and Cell-Specific KOs

Animal studies complied with the UK Home Office Guidance on the Operation of the Animals (Scientific Procedures) Act, 1986 and institutional guidelines. The generation of Nox2^fl/fl^ mice was commissioned from Genoway (France). The targeting construct was electroporated into 129sv embryonic stem cells. Recombinant clones were identified by polymerase chain reaction and Southern blotting. After successful targeting, the neomycin cassette was excised with the use of flanking Flippase Recognition Target sites. Clones were injected into C57BL/6 blastocysts. Heterozygous floxed mice obtained from germline chimeras were back-crossed >10 generations with C57BL/6 mice. For generation of cell-specific KO and littermate controls, Nox2^fl/fl^ females were crossed with male Tie2-Cre,^17^ LysM-Cre,^18^ or Cdh5-CreERT2^19^ transgenic mice. Inducible deletion of Nox2 in the Cdh5-CreERT2 model was achieved by tamoxifen treatment (40 mg/kg IP for 3 consecutive days). Adult male mice 8 to 16 weeks old with cell-specific Nox2 deletion were compared with Cre-negative Flox littermates.

### Polymerase Chain Reaction

Confirmation of cell-specific Nox2 deletion by polymerase chain reaction was based on the amplification of a 225–base pair (bp) product that is formed only after excision of exons 1 and 2 of the cybb gene (forward, GGAATTGAGTTGTAAGAATCAAATGAC; reverse, ATGATGTGTCCCAAATGTGC). Primer GGGGCTGAATGTCTTCCTCT was included in the reaction to detect the 467-bp wild-type Nox2 sequence.

Real-time reverse transcription–polymerase chain reaction with SYBR Green was used to quantify mRNA expression levels. ΔΔCt values were calculated with GAPDH used as denominator. Primer sequences were (forward and reverse) as follows: GAPDH, ATGACAACTTTGTCAAGCTCATTT and GGTCCACCACCCTGTTGCT; Nox2, ACTCCTTGGGTCAGCACTGG and GTTCCTGTCCAGTTGTCTTCG; p22^phox^, GCCCTCCACTTCCTGTT and GCAGATAGATCACACTGGCAAT; p40^phox^, CTGCTTTTCTGACTACCCACAG and AAGCTGCTCAAAGTCGCTCT; p47^phox^, GGACACCTTCATTCGCCATA and CTGCCACTTAACCAGGAACAT; p67^phox^, TTGAACCTGTCACACAGCAAT and CCAGCACACACACAAACCTT; superoxide dismutase (SOD)-1, GGACCTCATTTTAATCCTCACTCTAAG and GGTCTCCAACATGCCTCTCTTC; SOD2, CACACATTAACGCGCAGATCA and GGTGGCGTTGAGATTGTTCA; SOD3, ACACCTTAGTTAACCCAGAAATCTTTTC and GGGATGGATCTAGAGCATTAAGGA; and catalase, GCTGAGAAGCCTAAGAACGCAAT and CCCTTCGCAGCCATGTG.

### Immunoblotting

Snap-frozen aortic tissue was homogenized for immunoblotting. Primary antibodies were as follows: Nox2 (1:1000; BD Biosciences, Oxford, UK), p22^phox^ (1:1000; Santa Cruz), β-actin (1:4000; Sigma, UK), Nox4 (1:1000),^20^ endothelial NO synthase (eNOS; 1:1000; BD Biosciences). Actin (Sigma) was used as a loading control. Protein bands were visualized with enhanced chemiluminescence and quantified by densitometry.

### BP Measurement

BP telemeters (model TA11PA-C10, Data Sciences International) were implanted subcutaneously under isoflurane anesthesia, with a 1-week recovery period before measurements.^21^ Analyses were performed with Dataquest ART analysis software. AngII (1.1 mg·kg^−1^·d^−1^) was administered via subcutaneous osmotic minipumps (model 1002, Alzet, Cupertino, CA) implanted under 2% isoflurane.^21^ In some experiments, *N*^ω^-nitro-l-arginine methyl ester (L-NAME; Sigma-Aldrich, UK; 100 mg·kg^−1^·d^−1^) was administered in the drinking water.

### Magnetic Resonance Imaging

Magnetic resonance imaging was performed in prone mice on a 7-T horizontal scanner (Agilent Technologies, Varian Inc, Palo Alto, CA) under isoflurane anesthesia.^22^ Body temperature was maintained at 37°C, and heart rates were maintained >400 bpm. Temporally resolved dynamic short-axis images of the carotid arteries were acquired with a cine–fast low-angle shot sequence with ECG and respiratory gating. Endothelium-dependent relaxation in vivo was assessed with acetylcholine (18.8 mg/kg IP).^22^ Pixels encompassing the blood pool were clustered on the basis of signal intensity and the vessel wall borders. Images were analyzed in the end-systolic and end-diastolic phases with ClinicalVolumes segmentation software (King’s College London; www.clinicalvolumes.com).

### Other In Vivo Procedures

Echocardiography was performed with a Vevo 2100 system with a 40-MHz linear probe (VisualSonics, Inc) under 1.5% isoflurane anesthesia.^23^ Renal function was assessed in response to a short-term saline challenge (40 mL/kg 0.9% wt/vol saline, intraperitoneal injection). Animals were placed in an individual metabolic chamber (Tecniplast 3600M021) for 4 hours without access to food or water, and urine was collected at hourly intervals.^24^ Metabolites were analyzed on an Advia 2400 Chemistry System (Siemens AG, Germany).

### Ex Vivo Vascular Function

Isometric tension was quantified in descending thoracic aortic rings suspended in a Krebs buffer solution containing indomethacin (3 µmol/L) at 37°C, pH 7.4.^21^ Endothelium-dependent relaxation was assessed from the cumulative dose-response to acetylcholine of rings preconstricted to 70% of the maximal contraction to phenylephrine. In some experiments, rings were incubated with AngII (0.1 µmol/L for 4 hours) in the presence or absence of the superoxide scavenger MnTMPyP (10 µmol/L) before the addition of other vasoactive agents. Basal NO bioavailability was assessed from the response to a single dose of *N*-methyl-l-arginine (100 µmol/L) in rings preconstricted to 30% of the maximal phenylephrine response.

Vascular segments from mesenteric (second-order) arteries were studied in a tension myograph (Danish Myo Technology, Denmark) in Krebs buffer at 37°C.^25^ Endothelium-dependent relaxation was assessed from the cumulative dose response to acetylcholine in rings preconstricted with U46619 (0.1 μmol/L; Sigma).

### ROS Assays

High-performance liquid chromatography–based detection of dihydroxyethidium oxidation products was performed as described previously.^26^ Aortic segments were incubated with or without AngII (0.1 µmol/L for 3.5 hours at 37°C) before the addition of dihydroxyethidium (100 µmol/L) for 30 minutes at 37°C in the dark. Tissue was harvested in acetonitrile, sonicated, and centrifuged. Supernatants were dried under vacuum, and pellets were stored at −80°C. For analysis, samples were resuspended in 120 µL PBS/DTPA and injected into the high-performance liquid chromatography system. The superoxide-specific 2-hydroxyethidium signal was normalized to tissue weight.

ROS production in bone marrow and blood mononuclear cells was assessed by flow cytometry of cells loaded with dihydroxyethidium (10 µmol/L for 10 minutes at 37°C) after stimulation with phorbol 12-myristate 13-acetate (100 ng/mL) to activate the Nox2 oxidase complex.^27^ Bone marrow cells were harvested as described previously.^28^ Monocytes were isolated by Ficoll gradient centrifugation and CD11b Microbeads (Miltenyi Biotech, Germany).

### Vascular Morphology

In vivo perfusion fixation with 4% paraformaldehyde under pressure followed by paraffin embedding was used.^21^ Vascular media thickness and intima-media area were quantified in 6-µm sections stained with hematoxylin and eosin using Volocity software (Volocity version 5.0, Perkin Elmer, American Fork, UT).

Coronary microvascular endothelial cells were isolated from mouse hearts as described previously.^29^

### Flow Cytometry (Fluorescence-Activated Cell Sorter)

Quantitative analyses of leukocyte number and phenotype were performed by fluorescence-activated cell sorter on aortic tissue digests using an FACS CantoII instrument (BD Biosciences). In brief, animals under terminal anesthesia were perfused with saline through the left ventricle to eliminate circulating blood. The blood-free descending and abdominal aorta was digested in a mixture of collagenase IV, DNase, and hyaluronidase at 37°C for 30 minutes, followed by trituration and filtration through a 70-µm nylon mesh. Cell suspensions were washed and blocked with anti-CD16/CD32 antibodies before staining. Monocytes (CD45^+^CD11b^+^Ly6G^−^), macrophages (CD45^+^CD11b^+^F4/80^+^), neutrophils (CD45^+^CD11b^+^Ly6G^+^), T cells (CD45^+^TCRβ^+^), and B cells (CD45^+^CD19^+^) were identified. Zombie-Aqua dye (Biolegend) was used to identify dead cells before fixation with 1% paraformaldehyde. Fluorescence-minus-one–stained samples were used as negative controls. Data analysis was performed with FlowJo software (Tree Star Inc, Ashland, OR).

### NO Measurement by Electron Paramagnetic Resonance

Aortic NO formation was measured as described previously^30^ by electron paramagnetic resonance (EPR)–based spin trapping with iron-diethyldithiocarbamate [Fe(DETC)_2_] colloid with a Miniscope MS400 table-top X-band spectrometer (Magnettech, Berlin, Germany). In brief, aortas were stimulated either with calcium ionophore for measurement of eNOS-derived NO levels or with lipopolysaccharide to detect inducible NO synthase (iNOS)–derived NO formation. For lipopolysaccharide stimulation, freshly prepared aortas were cut into 3-mm rings and incubated with 10 μg/mL lipopolysaccharide in RPMI 1640 medium plus 10% FCS plus 1% penicillin/streptomycin for 21 hours at 37°C, 5% CO_2_. Afterward, rings were transferred to a 24-well plate filled with 1 mL Krebs-HEPES solution (pH 7.35, containing NaCl 99.01 mmol/L, KCl 4.69 mmol/L, CaCl_2_ 2.50 mmol/L, MgSO_4_ 1.20 mmol/L, NaHCO_3_ 25.0 mmol/L, K_2_HPO_4_ 1.03 mmol/L, Na-HEPES 20.0 mmol/L, D-glucose 11.1 mmol/L). For activation of eNOS, 10 µmol/L calcium ionophore (A12187, Sigma) was added to freshly prepared aortic rings in 1 mL Krebs-HEPES solution 2 minutes before Fe(DETC)_2_ spin trap addition. Then, 1 mL colloid Fe(DETC)_2_ was added to each well (0.4 mmol/L in PBS Ca^2+^/Mg^2+^) and incubated at 37°C, 10% CO_2_ for 1 hour. After incubation, aortic rings were snap-frozen in a 1-mL syringe, and recordings were performed at 77 K, with a Dewar flask. Instrument settings were as follows: B0 field=3300 G, sweep=110 G, sweep time=30 seconds, steps=4096, number pass=10, modulation=7000 mG, power=10 mW, and gain=9 E2. Levels of NO are expressed as intensity of signal (arbitrary units) per weight of wet sample.

### Statistics

All data are expressed as mean±SEM. Comparisons were made by Student *t* tests, 2-way ANOVA, or 2-way repeated measures ANOVA followed by Newman-Keuls post hoc tests as appropriate. Concentration-response curves were fitted with a sigmoid dose-response curve with fixed Hill slope (also known as 4-parameter logistic equation). Curves were compared by nonlinear regression analysis followed by the extra sum-of-squares *F* test. Data were analyzed with GraphPad Prism version 6 or SigmaStat version 3.5. Values of *P*<0.05 were considered significant.

## Results

### Tie2-Cre–Targeted Deletion of Nox2 in Mice In Vivo Reduces Basal BP

**Figure 1.**
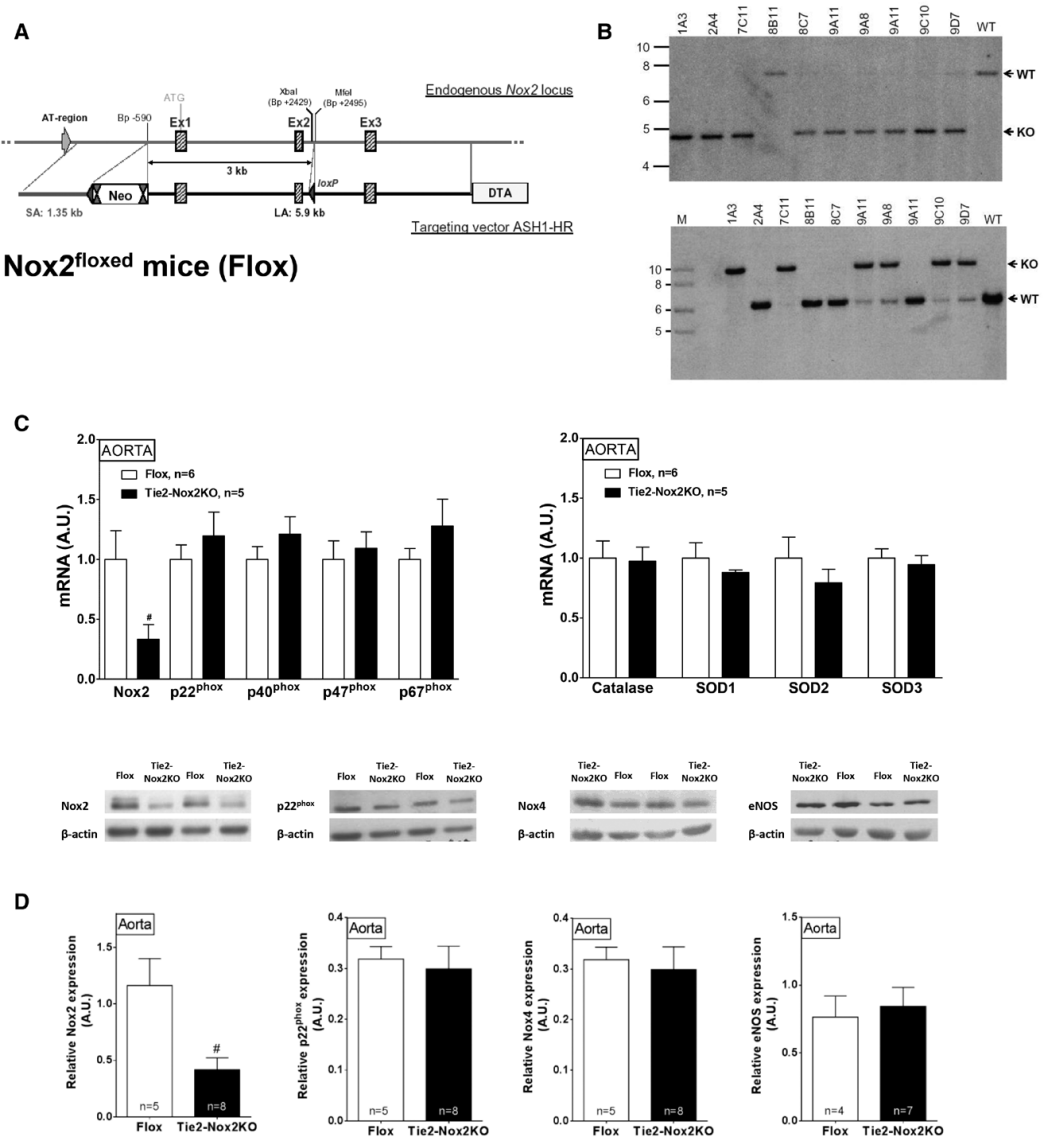
**Generation of Tie2-Nox2 knockout (KO) mice.** **A**, Targeting strategy for generation of Nox2^flox^ mice (Flox). The endogenous Nox2 locus is shown at the **top** and the targeting vector at the **bottom**. LoxP sites are represented by blue triangles; FRT sites, by double red triangles. After successful targeting, the neomycin (Neo) cassette was excised with the FRT sites. Cre-mediated recombination deletes a 3-kilobase fragment of Nox2, including the transcriptional initiation site and the first 2 exons. **B**, Southern blot analysis of genomic DNA from selected ES cell clones screened for 5′-homologous (**top**) and 3′-homologous (**bottom**) recombination. The clones 1A3 and 7C11 were used for blastocyst injection to generate Nox2^flox^ mice. WT indicates wild-type. **C**, mRNA levels of Nox2, accessory subunits, and antioxidant genes in Tie2-Nox2KO and Flox control aortas. **D**, Protein levels of Nox2, p22^phox^, Nox4, and endothelial nitric oxide synthase (eNOS) in Tie2-Nox2KO and control aortas. Representative immunoblots are shown above and mean data below. #*P*<0.05 vs Flox.

We first generated a mouse model with a “floxed” Nox2 allele on a C57Bl6 background such that Cre-mediated recombination deletes a 3-kilobase fragment of Nox2, including the transcription initiation site and the first 2 exons (Figure 1A and 1B). Homozygous floxed mice (Nox2^fl/fl^) were crossed with Tie-2 Cre mice to achieve endothelium-targeted deletion of Nox2 (Tie2-Nox2KO), although myelomonocytic cells may also be targeted with this promoter.^31^ Tie2-Nox2KO mice were born at the expected mendelian ratio and had no gross abnormalities up to 6 months of age. Body and major organ weights were similar between Tie2-Nox2KO and Flox littermates (eg, body weight, 26.8±0.3 versus 26.4±0.1 g at 10 weeks of age; n ≥10 per group). The aorta of Tie2-Nox2KO mice showed a significant reduction in Nox2 mRNA levels but no changes in the mRNA levels of p22^phox^, p47^phox^, p67^phox^, p40^phox^, SOD1 through SOD3, or catalase (Figure 1C). Nox2 mRNA levels were also substantially reduced in coronary microvascular endothelial cells from Tie2-Nox2KO mice compared with control (Figure IA in the online-only Data Supplement). Nox2 protein levels were significantly decreased in Tie2-Nox2KO mouse aorta compared with control Flox mice, but there were no differences in p22^phox^, Nox4, or eNOS protein levels (Figure 1D). A Tie2-driven deletion of Nox2 was therefore not accompanied by significant changes in antioxidant genes, Nox4, or eNOS.

**Figure 2.**
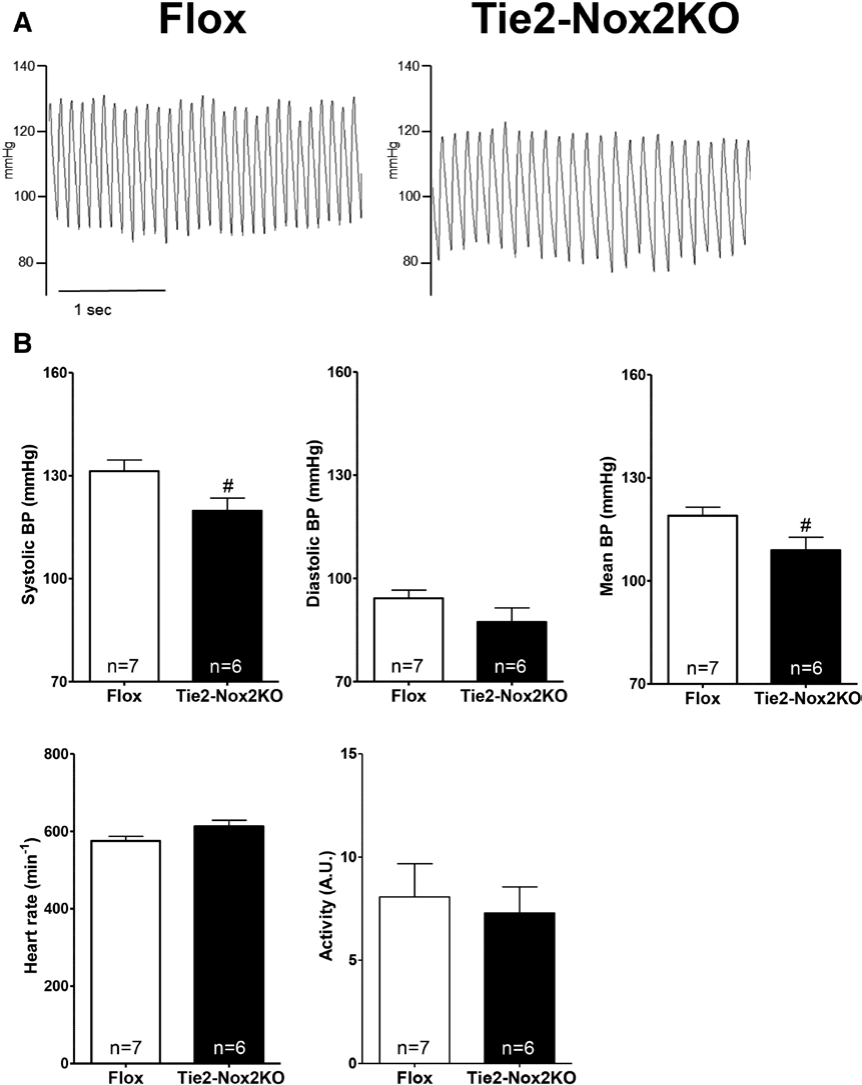
**Tie2-Nox2 knockout (KO) mice have reduced basal blood pressure.** **A**, Representative telemetric blood pressure (BP) traces in Tie2-Nox2KO mice and Flox controls. **B**, Mean data for BP, heart rate, and activity level. #*P*<0.05 vs Flox.

Assessment of ambulatory BP by telemetry revealed that Tie2-Nox2KO mice had a significantly lower basal systolic BP and mean BP than control Flox mice by ≈10 mm Hg (Figure 2A and 2B). There were no differences between groups in heart rate, activity levels, or cardiac structure and function assessed by echocardiography (Figure 2B and Figure IB in the online-only Data Supplement).

### Lower Basal BP in Tie2-Nox2KO Is Not Accounted for by Altered Renal Function, Vascular Remodeling, or Endothelium-Dependent Vasodilation

**Figure 3.**
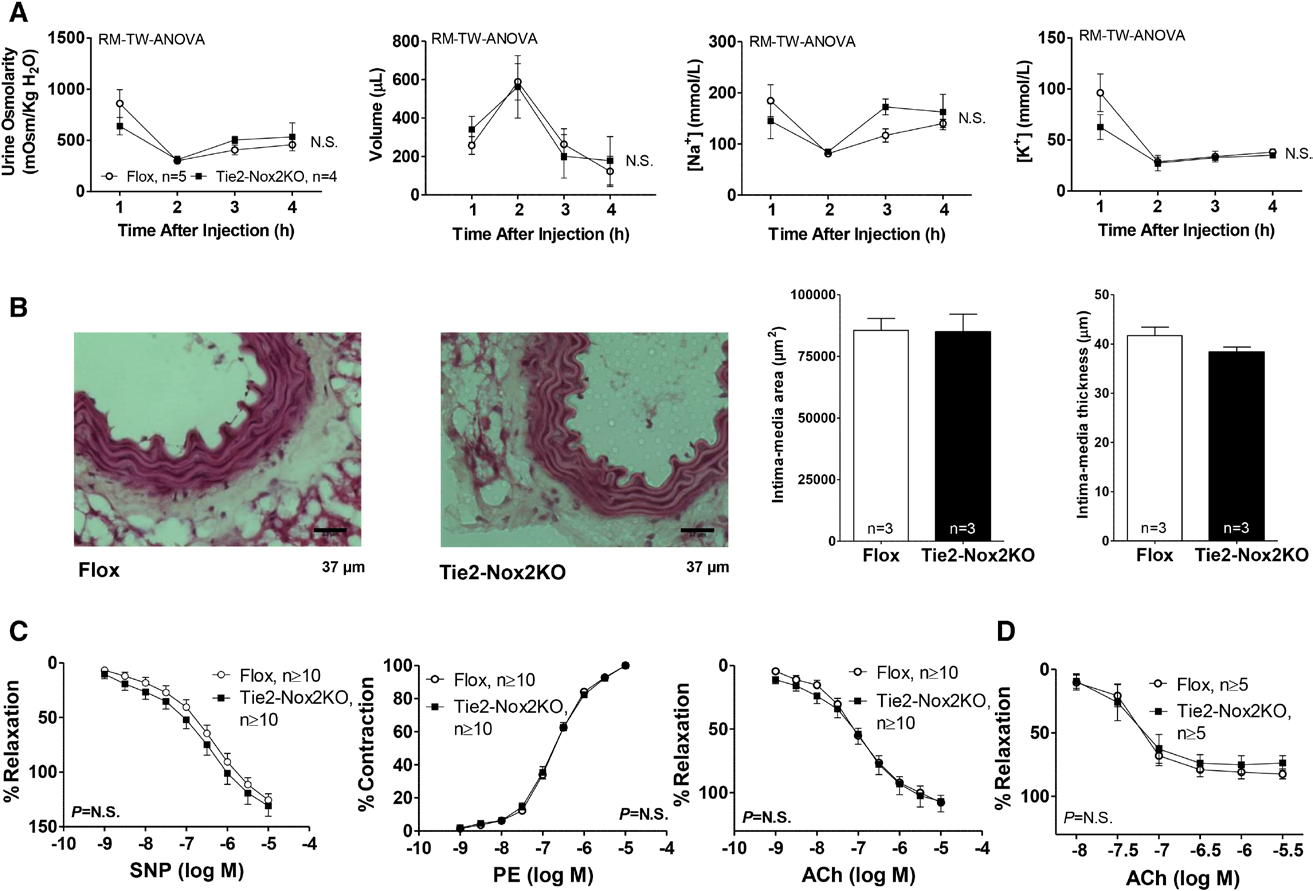
**Renal function, vascular remodeling, and in vitro vascular function in Tie2-Nox2 knockout (KO) mice.A**, Renal function assessed by response to a short-term saline challenge. Changes in urine osmolarity, volume, and sodium and potassium concentrations are shown. RM-TW-ANOVA indicates repeated-measures 2-way ANOVA; and N.S., not significant between groups. **B**, Representative histological sections of aortas from Tie2-Nox2KO and Flox mice (×40 magnification) and mean intima-media area and thickness. **C**, Concentration-response curves for response of aortic rings to the nitric oxide donor sodium nitroprusside (SNP), phenylephrine (PE), and acetylcholine (ACh). **D**, Concentration-response curves for response of mesenteric arteries to ACh.

There were no differences between Tie2-Nox2KO and control mice in urinary volume, electrolytes, or osmolarity in response to a short-term saline challenge (Figure 3A). To look for vascular remodeling as a basis for the lower BP, we quantified intima-media thickness and area in aortic sections, but this was also similar between groups (Figure 3B). Endothelium-dependent relaxation assessed from the vasodilator response to acetylcholine in isolated aortic rings was no different between Tie2-Nox2KO and control mice (Figure 3C). The vascular smooth muscle response to the NO donor sodium nitroprusside and the constrictor response to phenylephrine were similar in both groups. Acetylcholine-induced vasodilation in mesenteric arteries was also similar between groups (Figure 3D).

### Vascular and BP Responses of Tie2-Nox2KO Mice During AngII Stimulation

**Figure 4.**
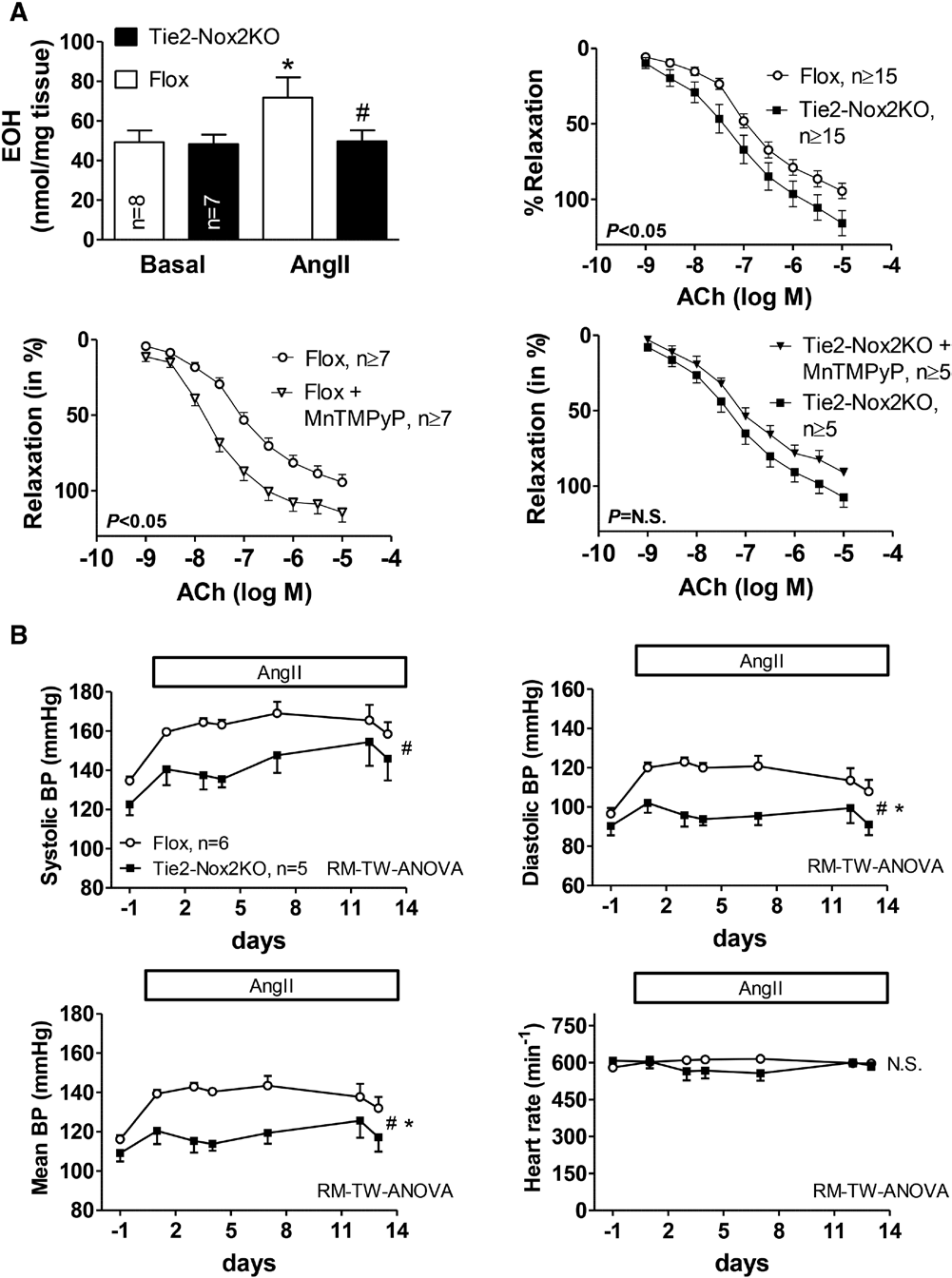
**Tie2-Nox2 knockout (KO) mice demonstrate blunted in vitro and in vivo responses to angiotensin II (AngII).** **A**, **Top Left**, Superoxide levels in Tie2-Nox2KO and Flox aortas at baseline and after AngII stimulation, assessed by high-performance liquid chromatography of the specific dihydroethidium oxidation product 2-hydroxyethidium (EOH). *P<0.05 vs control conditions (2-way-ANOVA). #*P*<0.05 vs Flox. **Top Right**, Effect of AngII pretreatment on vasodilation of aortic rings to acetylcholine (ACh). **Bottom**, Effect of MnTMPyP on ACh responses in AngII-treated rings from Flox mice (**left**) and Tie2-Nox2KO mice (**right**). **B**, Effect of long-term AngII infusion (1.1 mg·kg^−1^·d^−1^) on telemetric blood pressure (BP) and heart rate in Tie2-Nox2KO and Flox mice. RM-TW-ANOVA indicates repeated-measures 2-way ANOVA. *Significant interaction. #Significant difference between genotypes.

After exposure of aortic rings to AngII (0.1 µmol/L for 4 hours), the phenylephrine-induced vasoconstriction was similar in both groups, indicating comparable baselines. Acetylcholine-induced vasodilation was significantly greater in Tie2-Nox2KO than control mice (Figure 4A, top right). Aortic superoxide levels were similar in Tie2-Nox2KO and control aorta at baseline but were significantly higher in the control group after AngII treatment (Figure 4A, top left). In line with this, the superoxide scavenger MnTMPyP (10 µmol/L) improved endothelium-dependent relaxation in the control group but had no significant effect in Tie2-Nox2KO (Figure 4A, bottom). We also found that short-term NO synthase inhibition with *N*-methyl-l-arginine (100 µmol/L) induced greater vasoconstriction in AngII-treated Tie2-Nox2KO aortic rings than AngII-treated control rings (Figure IIA in the online-only Data Supplement), suggestive of a greater amount of bioactive NO in the former setting. In vivo, the hypertensive response observed in control mice with a 2-week infusion of AngII at 1.1 mg·kg^−1^·d^−1^ was significantly blunted in Tie2-Nox2KO animals, with no difference in the heart rate response (Figure 4B). Thus, the blunted hypertensive response to AngII observed in Tie2-Nox2KO mice may be attributable to a lower AngII-induced increase in endothelial superoxide, a consequent higher level of NO bioavailability, and a greater extent of endothelium-dependent vasorelaxation.

### Tie2-Nox2KO Mice Have Increased Basal NO Bioavailability In Vivo

**Figure 5.**
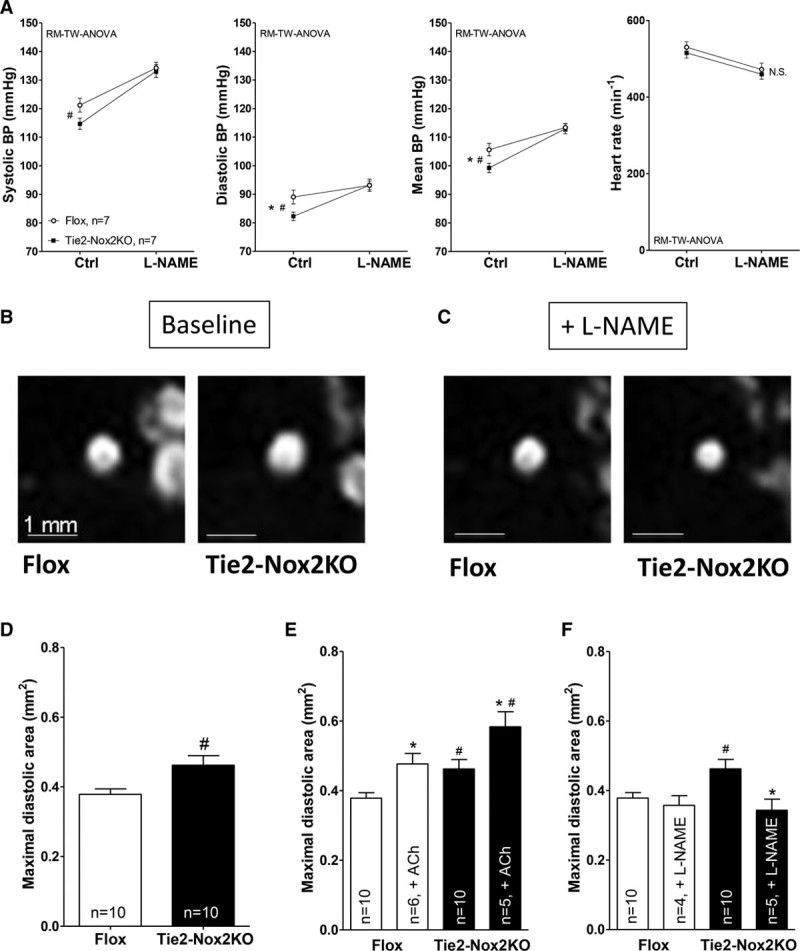
**Tie2-Nox2 knockout (KO) mice have increased basal nitric oxide (NO) bioavailability in vivo.** **A**, Blood pressure (BP) response to *N*^ω^-nitro-l-arginine methyl ester (L-NAME) in Tie2-Nox2KO and Flox mice, assessed by telemetry. *Significant interaction as tested by repeated-measures 2-way ANOVA (RM-TW-ANOVA). #Significant difference between genotypes. **B** and **C**, Representative magnetic resonance images of the carotid artery in Tie2-Nox2KO and Flox mice at baseline (**B**) and after L-NAME treatment (**C**). Scale bar, 1 mm. **D** through **F**, Mean data for diastolic area of the carotid artery at baseline (**D**), after acetylcholine (ACh) treatment (**E**), and after L-NAME treatment (**F**). *P<0.05 vs control conditions (TW-ANOVA). #*P*<0.05 vs Flox.

Although basal ex vivo endothelium-dependent vascular function was similar between Tie2-Nox2KO and control mice, it is possible that this might be different in vivo and that an increase in NO bioavailability caused by a reduction in Nox2-derived superoxide may account for the lower basal BP in Tie2-Nox2KO. We tested this idea by assessing the ambulatory BP response to the NO synthase inhibitor L-NAME (100 mg·kg^−1^·d^−1^ orally for 2 days). The hypertensive response to L-NAME was found to be significantly greater in Tie2-Nox2KO than control mice, such that BP levels were similar in the 2 groups after L-NAME treatment (Figure 5A). This finding suggests that in vivo NO bioavailability at baseline may be higher in Tie2-Nox2KO than control mice. To assess whether L-NAME–mediated changes in BP involved the vasculature, we used magnetic resonance imaging–based measurement of resistance artery caliber in vivo (by imaging the carotid artery). The carotid artery luminal area in Tie2-Nox2KO was significantly higher than in matched control mice at baseline (0.46±0.03 versus 0.38±0.02 mm^2^; n=10 each; *P*<0.05; Figure 5B and 5D), consistent with basal vasodilatation. Acetylcholine induced a similar extent of vasodilatation in both groups of mice (Figure 5E), consistent with the results in ex vivo vessels. After L-NAME treatment, however, Tie2-Nox2KO carotid arteries constricted to a greater extent than carotid arteries in control mice, such that luminal vessel diameters were now similar in both groups (Figure 5C and 5F). These results are consistent with the notion that a higher in vivo NO bioavailability may increase basal resistance vessel caliber in Tie2-Nox2KO mice.

### Contrasting Roles of Endothelial Versus Myeloid Immune Cell Nox2 in BP Regulation

**Figure 6.**
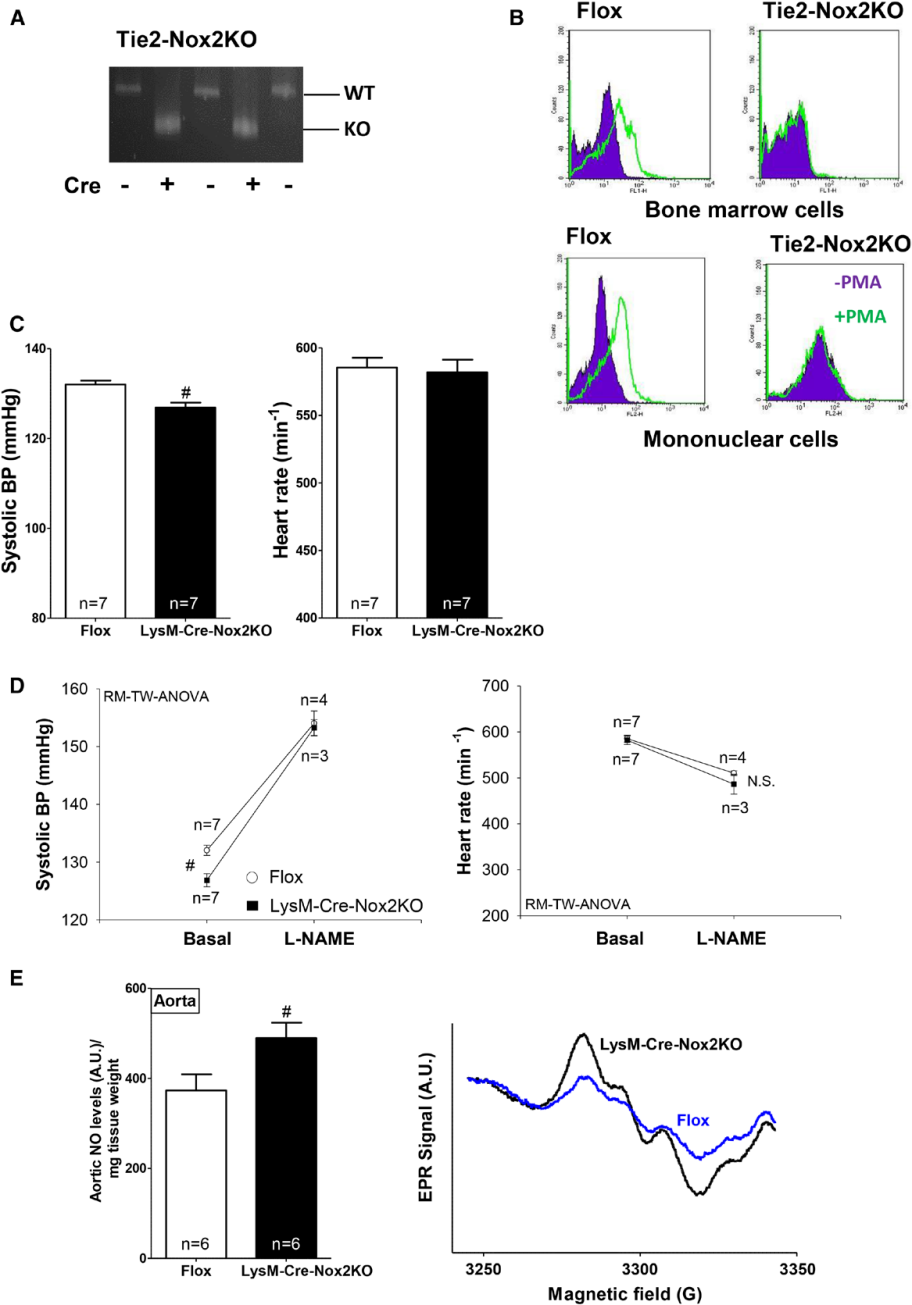
**LysM-Cre-Nox2 knockout (KO) mice have reduced basal blood pressure (BP) and increased vascular nitric oxide (NO) bioavailability.** **A**, Cre-mediated recombination in bone marrow–derived cells from Tie2-Nox2KO mice. WT indicates wild-type. **B**, Functionally deficient reactive oxygen species production in phorbol ester (PMA)–stimulated bone marrow cells and circulating mononuclear cells from Tie2-Nox2KO mice, assessed by flow cytometry in cells loaded with dihydroethidium. **C**, Reduced basal BP (telemetry) in LysM-Cre-Nox2KO mice. #*P*<0.05 vs Flox. **D**, In vivo response to *N*^ω^-nitro-l-arginine methyl ester (L-NAME) in LysM-Cre-Nox2KO mice and controls. #Significance vs Flox as tested by repeated-measures 2-way ANOVA (RM-TW-ANOVA). **E**. Increased aortic NO levels in LysM-Cre-Nox2KO under basal conditions, as assessed by electron paramagnetic resonance (EPR). Representative EPR spectra are shown on the **right**. #*P*<0.05 vs Flox.

The data presented so far suggest that the lower basal BP in Tie2-Nox2KO animals involves an NO-dependent mechanism but cannot be accounted for by altered endothelial function. Because Tie2 is expressed not only in endothelial cells but also in myelomonocytic cells,^31^ we considered the possibility that the results observed in Tie2-Nox2KO mice might be related to Nox2 deletion in myeloid cells. Indeed, Tie2-Nox2KO mice showed clear evidence of Cre-mediated recombination in bone marrow–derived cells (Figure 6A). Furthermore, Tie2-Nox2KO bone marrow cells and circulating mononuclear cells displayed functionally deficient Nox2-derived ROS production (Figure 6B).

To investigate whether Nox2 in myeloid cells contributes to the effects on basal BP, we next generated a myeloid-specific Nox2KO model using a LysM-Cre mouse line that targets myelomonocytic cells^18^ (Figure IIB in the online-only Data Supplement). LysM-Cre-Nox2KO mice showed no obvious gross phenotype and had comparable amounts of myeloid inflammatory cells in the vessel wall (Table I in the online-only Data Supplement). However, ambulatory BP monitoring by telemetry revealed a significant reduction in basal systolic BP compared to control Flox mice, similar to that observed in Tie2-Nox2KO animals, with no alteration in heart rate (Figure 6C). To assess whether this reduction in basal BP was related to increased in vivo NO bioavailability, LysM-Cre-Nox2KO mice and controls were treated with L-NAME. Indeed, we found that after L-NAME treatment, which had hypertensive effects in both groups of mice, the systolic BP was similar in LysM-Cre-Nox2KO and controls (Figure 6D). To more directly assess basal vascular NO levels, we performed EPR with NO-Fe(DETC)_2_ spin trapping (Figure 6E). Aortic NO levels were significantly higher in LysM-Cre-Nox2KO mice than controls, consistent with the higher NO bioavailability in vivo (Figure 6D). It is interesting to note that although basal aortic NO levels were increased in LysM-Cre-Nox2KO, iNOS-derived NO formation as assessed after lipopolysaccharide stimulation was lower than in controls (Figure IIIA in the online-only Data Supplement). We also studied the response of LysM-Cre-Nox2KO mice to AngII infusion. In contrast to the differences in basal BP, the hypertensive response to AngII was similar in LysM-Cre-Nox2KO and control mice (Figure IIIB in the online-only Data Supplement), indicating that myeloid cell Nox2 does not appear to modulate AngII-dependent hypertension.

**Figure 7.**
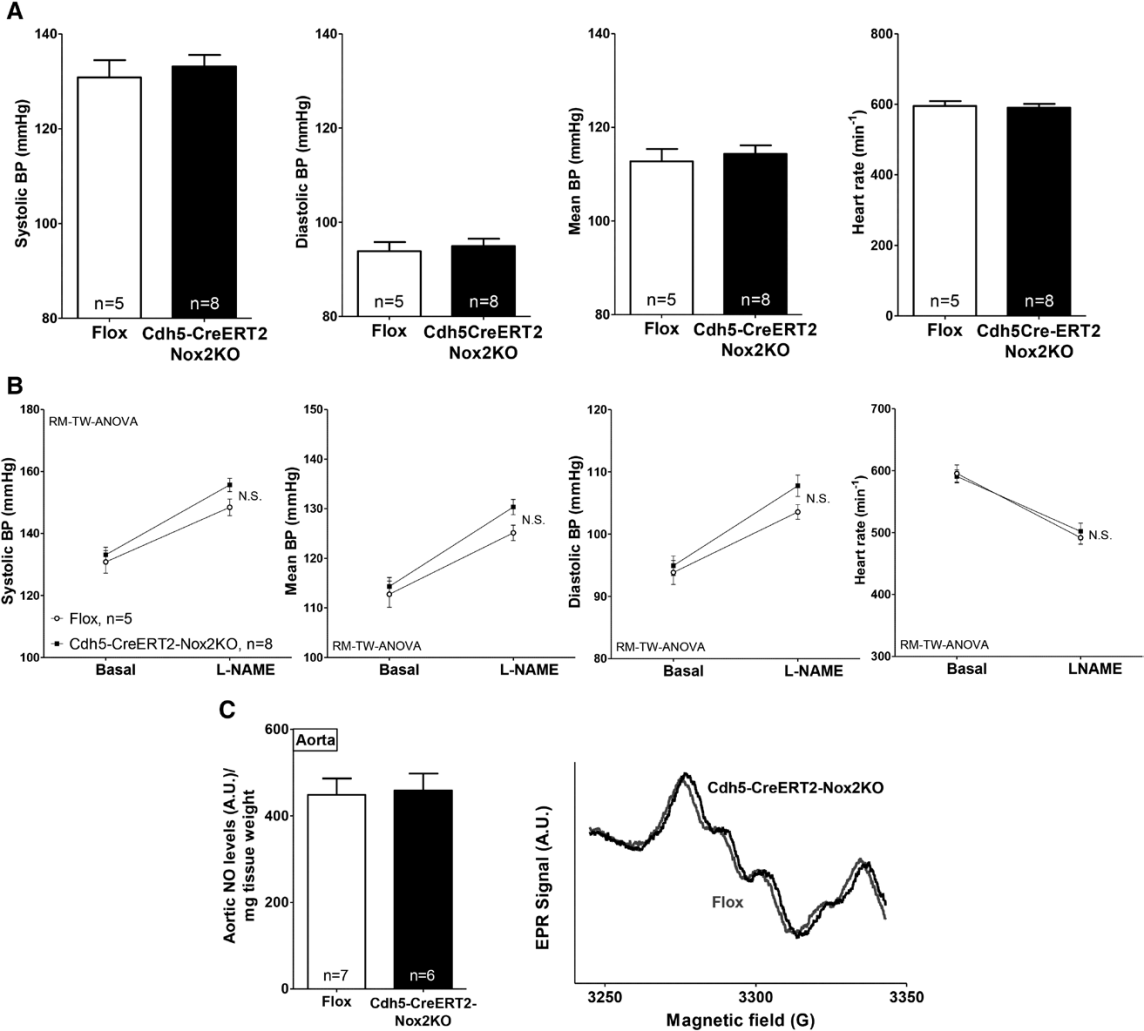
**Cdh5-CreERT2-Nox2 knockout (KO) mice have unaltered basal blood pressure (BP).** **A**, Telemetric BP and heart rate at baseline. **B**, BP responses to *N*^ω^-nitro-l-arginine methyl ester (L-NAME) in Cdh5-CreERT2-Nox2KO and Flox mice. L-NAME increased BP in both groups, but there was no significant difference between groups by repeated-measures 2-way ANOVA (RM-TW-ANOVA). **C**, Comparable aortic nitric oxide (NO) levels in Cdh5-CreERT2-Nox2KO and controls under basal conditions, as assessed by electron paramagnetic resonance (EPR). Representative EPR spectra are shown on the **right**.

To elucidate the specific role of Nox2 in the endothelium, we then generated an inducible endothelial Nox2KO model using a Cdh5-CreERT2 driver line.^19^ In this model, endothelium-specific Nox2 deletion was achieved in adult mice by tamoxifen treatment (Figure IIC in the online-only Data Supplement). Tamoxifen treatment had no effect on cardiac function (Figure IID in the online-only Data Supplement). In contrast to LysM-Cre-Nox2KO mice, Cdh5-CreERT2-Nox2KO animals showed no difference in basal BP compared with matched controls as assessed by ambulatory telemetry (Figure 7A). The hypertensive response to L-NAME was also similar in both groups (Figure 7B), suggesting a comparable NO bioavailability under basal conditions in this model. The quantification of basal aortic NO levels by EPR confirmed that they were similar between groups (Figure 7C). AngII-induced hypertension, however, was significantly attenuated in Cdh5-CreERT2-Nox2KO mice (Figure IIIC in the online-only Data Supplement), pointing to endothelial Nox2 as a crucial player in AngII-mediated hypertension.

## Discussion

This study provides significant new insights into the role of Nox2 in the regulation of BP. With the use of several novel cell-specific Nox2KO models, we were able to dissect out and distinguish between the effects of myelomonocytic cell Nox2 on basal BP and those of endothelial cell Nox2 on AngII-induced hypertension. We accordingly identified cell-specific and context-specific roles for Nox2 in BP regulation that indicate that the roles of ROS are highly complex not only among different ROS sources but also for the same source in different cell types.

An important and unexpected initial finding was that Tie2-Nox2KO mice, which we generated as a model of endothelium-specific Nox2 deletion, exhibited significantly lower basal BP compared with matched controls. Although some previous studies have reported a lower BP in global Nox2KO mice,^32,33^ it was not immediately obvious why the deletion of endothelial Nox2 should affect basal BP. For example, previous work from our group^21^ and others^34^ showed that endothelium-specific overexpression of Nox2 had no effect on basal BP. Compensatory changes in other enzymes such as the antioxidants SOD1 through SOD3 and catalase, eNOS, or Nox4 could conceivably be involved (and Nox4 was previously linked to lower BP^26^), but we found no differences in the levels of these enzymes between Tie2-Nox2KO and control mice. The investigation of ex vivo endothelium-dependent function in Tie2-Nox2KO mouse aorta and mesenteric artery did not reveal any enhancement of relaxation, in line with the finding that basal vascular O_2_^−^ production was unaltered. We also found no changes in cardiac or renal function and no evidence of structural vascular remodeling to explain the lower BP. Nevertheless, the lower BP in Tie2-Nox2KO mice was related to an enhanced in vivo NO bioavailability as indicated by the normalization of the BP difference on treatment with L-NAME. Furthermore, in vivo assessment of resistance vessel function by magnetic resonance imaging revealed that Tie2-Nox2KO mice had a pronounced vasodilatation under basal conditions that could be reversed by L-NAME treatment but showed no evidence of altered endothelium-dependent relaxation. Taken together, these findings led us to consider the possibility that nonendothelial Nox2-containing cells may be involved in the effect on basal BP. In this regard, it is known that Tie2 is expressed not only in endothelial cells but also in certain hematopoietic cells, notably monocytic cells.^31,35^ Indeed, we confirmed that the Tie2-Cre approach not only targeted endothelial cells but also induced functional Nox2 deletion in myelomonocytic cells.

The generation of novel LysM-Cre-Nox2KO and Cdh5-CreERT2-Nox2KO mice then allowed us to establish that the reduction in basal BP was in fact related to deletion of Nox2 from myelomonocytic cells rather than endothelial cells, with an associated increase in in vivo NO bioavailability. Using EPR with NO-Fe(DETC)_2_ spin trapping, we found that the aortas of LysM-Cre-Nox2KO mice had increased NO levels, pointing to the vasculature as the likely site of action of myelomonocytic cells with disrupted Nox2. Conversely, Cdh5-CreERT2-Nox2KO aortas had unaltered vascular NO levels under basal conditions. It should be noted that the magnitude of difference in NO levels observed in aorta cannot necessarily be extrapolated to the resistance vasculature, which is the more important vascular site for BP regulation. The effect of myelomonocytic cells in the vasculature is most likely to reflect the inactivation of endothelium-derived NO by myeloid Nox2-derived superoxide, before the NO can affect vascular smooth muscle cell relaxation. When myelomonocytic Nox2 is disrupted, the levels of bioactive NO would accordingly rise. Consistent with this idea, we could demonstrate the presence of myelomonocytic cells in the aortas of both LysM-Cre-Nox2KO and control mice but with no difference in the number of cells between groups. Myelomonocytic cells could in principle produce NO from iNOS, but we found that iNOS-derived NO (after lipopolysaccharide stimulation) was decreased in LysM-Cre-Nox2KO mice, consistent with previous reports that Nox-dependent signaling can increase iNOS expression,^36^ making it unlikely that myeloid cell iNOS plays a role in the observed changes in basal BP. The reason the deletion of only myelomonocytic Nox2 but not endothelial Nox2 affects basal BP is most likely that the abundance of Nox2 is much lower in endothelial cells than myeloid cells.^37^ We did note, however, that the reduction in basal BP was slightly higher in the Tie2-Nox2KO mice (in which both myeloid and endothelial cell Nox2 is deleted) than in LysM-Cre-Nox2KO. This could indicate that the effects of myelomonocytic Nox2KO may be enhanced by the concomitant knockout of endothelial cell Nox2. The present results are, to the best of our knowledge, the first indication that myelomonocytic cell Nox2 modulates basal BP.

AngII is a potent activator of both Nox1 and Nox2, and an impact of Nox enzymes on AngII-induced hypertension has been documented in many previous studies, as discussed earlier. However, the specific roles of different Nox isoforms and different cell types have remained unclear. Quite strong evidence supports a role for vascular smooth muscle Nox1 in AngII-induced hypertension, involving changes in vascular remodeling.^10,11^ The cell-specific role of Nox2 is unclear, but this a pertinent question because Nox2 is expressed in endothelial cells, cardiomyocytes, fibroblasts, certain vascular smooth muscle cells, and inflammatory/immune cells.^6^ Nox2 is involved in the genesis of endothelial dysfunction in diverse models,^6,7^ and previous studies from our laboratory^21^ and others^32^ found that endothelium-targeted overexpression of Nox2 enhanced AngII-induced hypertension. However, the role of endogenous endothelial Nox2 was not established in those studies. The present results in Cdh5-CreERT-Nox2KO mice and Tie2-Nox2KO mice clearly establish that endogenous endothelial Nox2 augments AngII-induced hypertension, at least in the relatively short term (2 weeks). It is interesting to note that AngII-induced hypertension was comparable in LysM-Cre-Nox2KO and respective control mice, indicating that constitutive deficiency of Nox2 in myelomonocytic cells is apparently not important in this setting. On the other hand, previous data suggest that T-cell Nox2^15^ and Nox2 in the subfornical organ,^12,13^ and possibly Nox2 in renal afferent arterioles,^14^ also modulate AngII-induced hypertension. Furthermore, it was also shown that mice can be protected from arterial hypertension when LysM-positive cells are depleted before the AngII infusion is begun and that BP can be restored by adoptive transfer of Nox2-competent monocytes into these mice.^16^ Taken together, these data indicate complex roles during AngII-induced hypertension for Nox2 in multiple cell types, some that involve altered NO bioavailability and others that may involve Nox2-dependent redox signaling events. The endothelium-dependent effects of Nox2 defined in the present study appear to involve an increased inactivation of NO by ROS, which affects endothelium-dependent relaxation, as suggested by our ex vivo studies, but could potentially also affect other NO-dependent functions.

The major new finding of this study is the potential for myelomonocytic cells to affect basal BP in a reversible manner. Although the absolute change in basal BP is modest, its magnitude is similar to that of changes that would be considered clinically or prognostically significant, for example, in hypertension. From a pathophysiological perspective, it is interesting to speculate whether the effects of these cells on BP might be enhanced in disease settings where monocytes are activated, for example, inflammatory conditions. In this regard, patients with chronic granulomatous disease, who have functionally deficient Nox2 activity, might be of particular interest. More broadly, the present results suggest the potential for different disease conditions to alter BP through Nox2-dependent effects in distinct cell types, that is, myeloid cells or endothelial cells. In terms of renin-angiotensin-aldosterone system–dependent hypertension, Nox2 inhibition would be anticipated to be beneficial, and combined Nox1/Nox2 inhibitors could be of particular value given the effects of both isoforms to increase BP.

## Conclusions

This study identifies distinct effects of myeloid cell Nox2 and endothelial cell Nox2 on basal and AngII-dependent BP, respectively, and suggests that Nox2 may be a master regulator of BP.

## Sources of Funding

These studies were supported by the British Heart Foundation (CH/1999001/11735), the German Cardiac Society, the Deutsche Forschungsgemeinschaft (SA 3282/1–1), and a Fondation Leducq Transatlantic Network of Excellence. Dr Schnelle was supported by a Deutsche Forschungsgemeinschaft Joint PhD Studentship (IRTG1816).

## Disclosures

None.

## Supplementary Material

## Clinical Perspective

### What Is New?

- NADPH oxidases generate superoxide and are implicated in the pathogenesis of hypertension.
- The NADPH oxidase Nox2 isoform is expressed in several cell types (such as endothelial cells and inflammatory cells), but exactly how it affects blood pressure (BP) is unclear.
- This study uses novel gene-modified mouse models to show that Nox2 in myeloid cells modulates basal BP, whereas endothelial cell Nox2 is involved in angiotensin II–dependent hypertension.
- This is the first demonstration that myeloid cell Nox2 regulates basal BP.

### What Are the Clinical Implications?

- The finding that Nox2 in different cell types has distinct effects on BP suggests that different disease conditions may alter BP through effects of Nox2 in distinct cell types.
- It is conceivable that the effects of myeloid cells on basal BP may be enhanced in inflammatory settings where these cells are more activated.
- On the other hand, endothelial cell Nox2 activation may be more relevant to renin-angiotensin system–dependent hypertension.
- The present results are relevant to the design of novel therapeutic approaches for hypertension by targeting NADPH oxidases.
