# Supplementary material for: Distinct Regulatory Effects of Myeloid Cell and Endothelial Cell NAPDH Oxidase 2 on Blood Pressure
Source: Circulation. 2017 May 30;135(22):2163–77. doi: 10.1161/CIRCULATIONAHA.116.023877 (PMC5444427; doi:10.1161/CIRCULATIONAHA.116.023877)
Supplement: Supplementary file 1 [file cir-135-2163-s001.pdf]

SUPPLEMENTAL MATERIAL

Supplementary Table 1. Aortic myelo-monotic and lymphatic cells in Flox control and LysM-Cre-Nox2KO mice.

	**Flox control**		**LysM-Cre Nox2KO**		
**Cell type**	**Number of cells / 10 mg aortic tissue**	**n**	**Number of cells 10 / mg aortic tissue**	**n**	***P***
$CD45^+CD11b^+$ $Ly6G^-$ (Monocytes)	100 ± 60	4	120 ± 20	5	N.S.
$CD45^+CD11b^+$ $F4/80^+$ (Macrophages)	3.5 ± 2.2	4	8.1 ± 1.3	5	N.S.
$CD45^+CD11b^+$ $Ly6G^+$ (Neutrophils)	540 ± 230	5	580 ± 80	5	N.S.
$CD45^+TCR\beta^+$ (T-Lymphocytes)	790 ± 340	5	810 ± 110	5	N.S.
$CD45^+CD19^+$ (B-Lymphocytes)	640 ± 310	5	640 ± 90	5	N.S.

Data are mean±SEM.

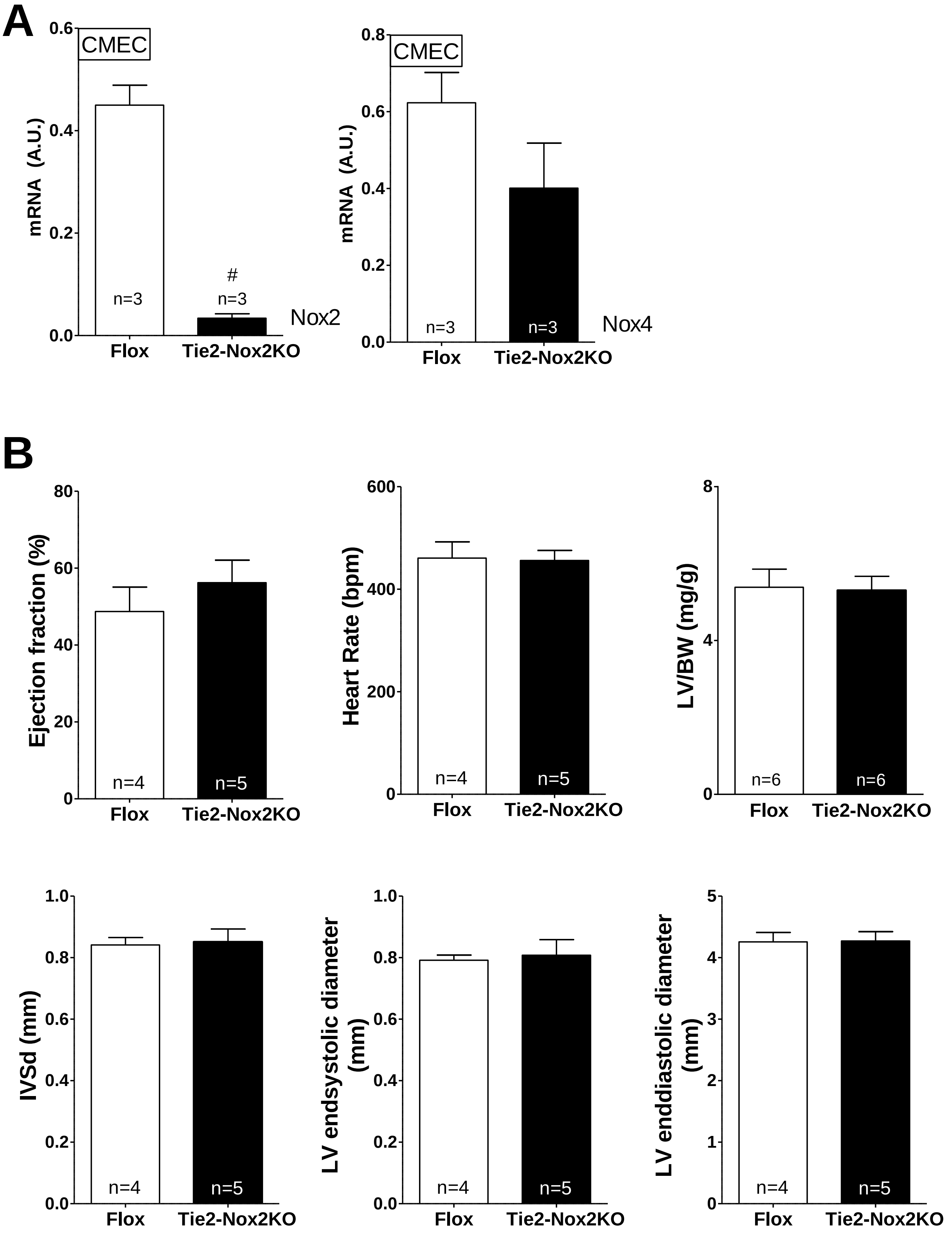
A
CMEC
mRNA (A.U.)
0.0
0.2
0.4
0.6
n=3
#
n=3
Nox2
Flox
Tie2-Nox2KO
CMEC
mRNA (A.U.)
0.0
0.2
0.4
0.6
0.8
n=3
n=3
Nox4
Flox
Tie2-Nox2KO
B
Ejection fraction (%)
0
20
40
60
80
n=4
n=5
Flox
Tie2-Nox2KO
Heart Rate (bpm)
0
200
400
600
n=4
n=5
Flox
Tie2-Nox2KO
LV/BW (mg/g)
0
4
8
n=6
n=6
Flox
Tie2-Nox2KO
IVSd (mm)
0.0
0.2
0.4
0.6
0.8
1.0
n=4
n=5
Flox
Tie2-Nox2KO
LV endsystolic diameter (mm)
0.0
0.2
0.4
0.6
0.8
1.0
n=4
n=5
Flox
Tie2-Nox2KO
LV enddiastolic diameter (mm)
0
1
2
3
4
5
n=4
n=5
Flox
Tie2-Nox2KO

Suppl. Fig. 1

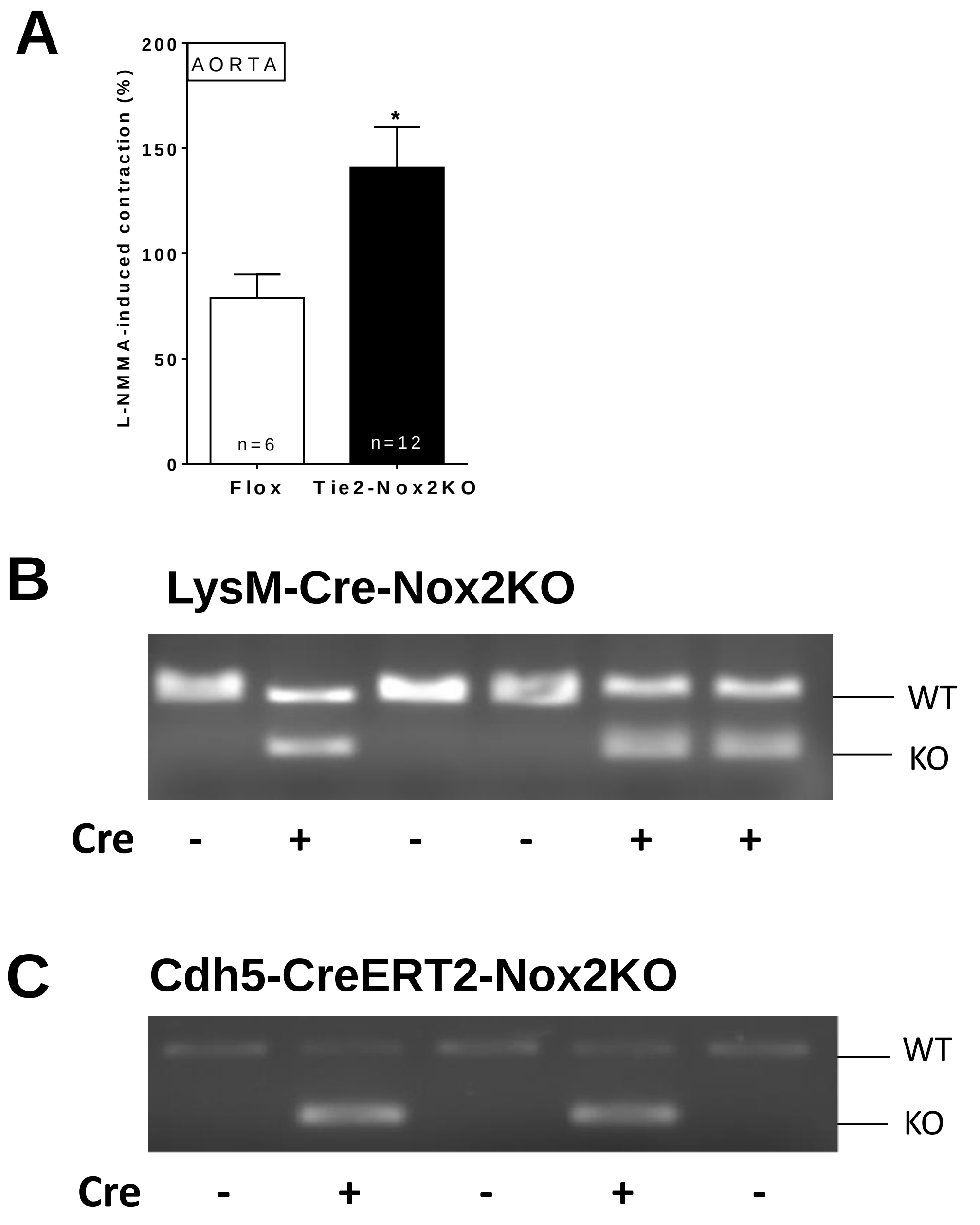
A
AORTA
L-NMMA-induced contraction (%)
200
150
100
50
0
*
n=6
n=12
Flox
Tie2-Nox2KO
B
LysM-Cre-Nox2KO
WT
KO
Cre - + - - + +
C
Cdh5-CreERT2-Nox2KO
WT
KO
Cre - + - + -

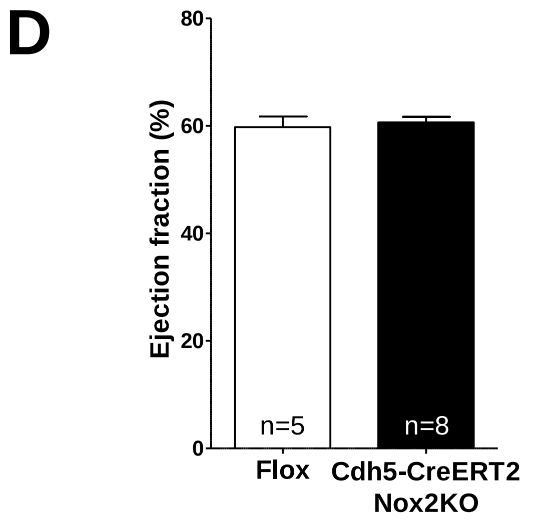
D
Ejection fraction (%)
80
60
40
20
0
n=5
n=8
Flox
Cdh5-CreERT2
Nox2KO

Suppl. Fig. 2

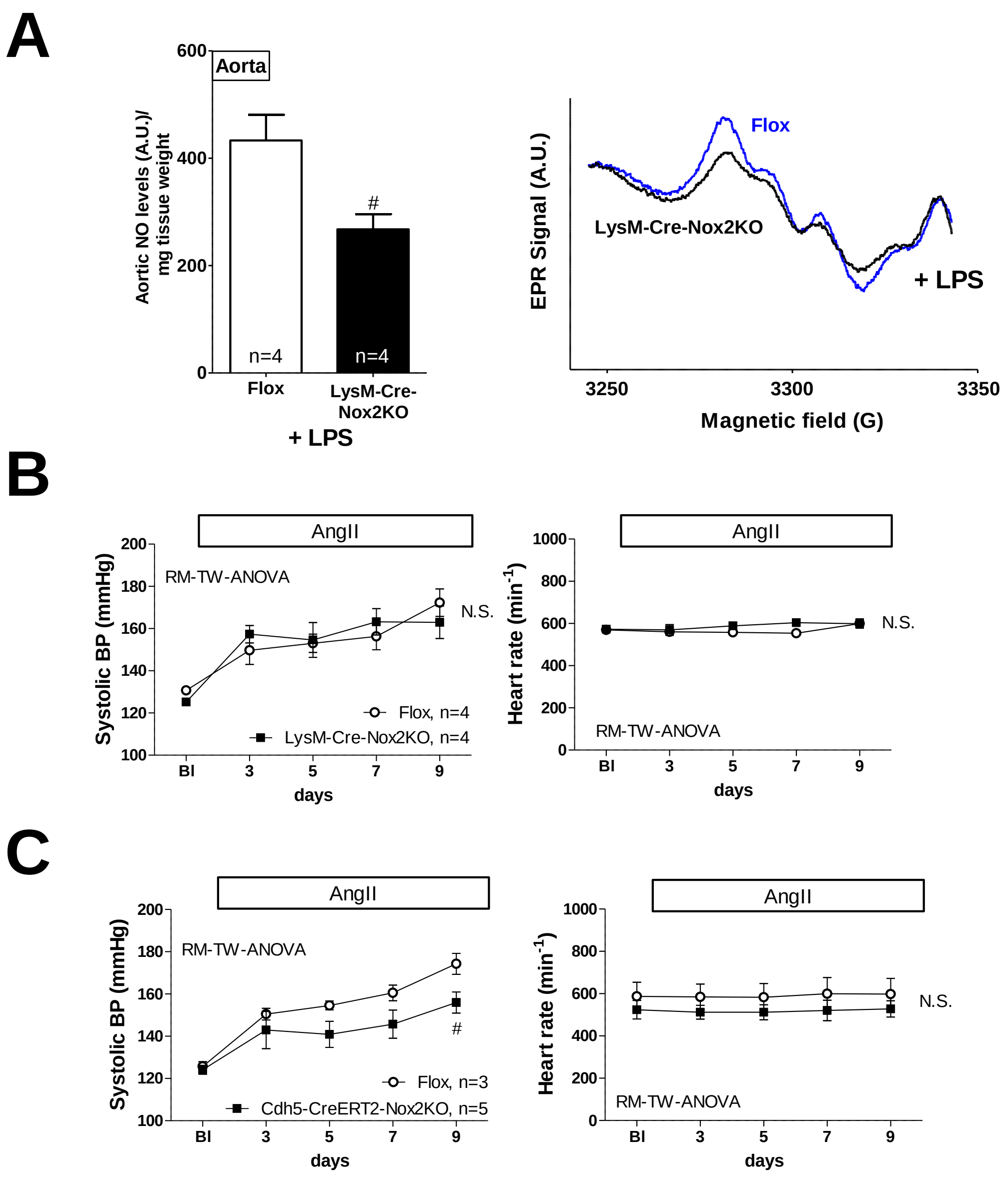
A
B
C
600
400
200
0
Aorta
Aortic NO levels (A.U.)/ mg tissue weight
#
n=4
n=4
Flox
LysM-Cre-Nox2KO
+ LPS
EPR Signal (A.U.)
Flox
LysM-Cre-Nox2KO
+ LPS
3250
3300
3350
Magnetic field (G)
AngII
Systolic BP (mmHg)
RM-TW-ANOVA
N.S.
Flox, n=4
LysM-Cre-Nox2KO, n=4
Bl
3
5
7
9
days
Heart rate (min-1)
AngII
N.S.
RM-TW-ANOVA
days
AngII
Systolic BP (mmHg)
RM-TW-ANOVA
#
Flox, n=3
Cdh5-CreERT2-Nox2KO, n=5
days
AngII
Heart rate (min-1)
N.S.
RM-TW-ANOVA
days

Suppl. Fig. 3

Supplementary Figure 1. Coronary microvascular endothelial cell mRNA levels and cardiac echocardiography.

A. mRNA levels of Nox2 and Nox4 in coronary microvascular endothelial cells (CMEC). $^{\#}$, *P*<0.05 vs. Flox. **B.** Echocardiographic parameters of cardiac structure and function, and left ventricle/body weight ratio (LV/BW). IVSD, interventricular septal diameter.

Supplementary Figure 2. Generation of LysM-Cre-Nox2KO and Cdh5-CreERT2-Nox2KO mice.

A. Magnitude of L-NMMA-induced constriction in AngII-treated aortic rings from Tie2-Nox2KO mice and Flox controls. *, *P*<0.05 vs. Flox. **B.** Cre-mediated recombination in Cre^{+} bone marrow cells from LysM-Cre-Nox2KO mice. **C.** Cre-mediated recombination in Cre^{+} lung tissue from Cdh5-CreERT2-Nox2KO mice. **D.** Left ventricular ejection fraction in Cdh5-CreERT2-Nox2KO cf. Flox mice following Tamoxifen treatment.

Supplementary Figure 3. Hypertensive response to AngII in LysM-Cre-Nox2KO and Cdh5-CreERT2-Nox2KO mice.

A. Reduced aortic iNOS-derived NO formation in LysM-Cre-Nox2KO after LPS stimulation. $^{\#}$, *P*<0.05 vs. Flox. Representative EPR spectra shown to the right. **B.** *In vivo* response to AngII infusion in LysM-Cre-Nox2KO mice and Flox control. **C.** *In vivo* response to AngII infusion in Cdh5-CreERT2-Nox2KO mice and respective control. RM-TW-ANOVA = repeated measures two-way ANOVA. $^{\#}$, *P*<0.05 vs. Flox control.
